# From Food Insufficiency towards Trade Dependency: A Historical Analysis of Global Food Availability

**DOI:** 10.1371/journal.pone.0082714

**Published:** 2013-12-18

**Authors:** Miina Porkka, Matti Kummu, Stefan Siebert, Olli Varis

**Affiliations:** 1 Water & Development Research Group, Aalto University, Espoo, Finland; 2 Institute of Crop Science and Resource Conservation (INRES), University of Bonn, Bonn, Germany; New York State Museum, United States of America

## Abstract

Achieving global food security is one of the major challenges of the coming decades. In order to tackle future food security challenges we must understand the past. This study presents a historical analysis of global food availability, one of the key elements of food security. By calculating national level dietary energy supply and production for nine time steps during 1965–2005 we classify countries based on their food availability, food self-sufficiency and food trade. We also look at how diets have changed during this period with regard to supply of animal based calories. Our results show that food availability has increased substantially both in absolute and relative terms. The percentage of population living in countries with sufficient food supply (>2500 kcal/cap/d) has almost doubled from 33% in 1965 to 61% in 2005. The population living with critically low food supply (<2000 kcal/cap/d) has dropped from 52% to 3%. Largest improvements are seen in the MENA region, Latin America, China and Southeast Asia. Besides, the composition of diets has changed considerably within the study period: the world population living with high supply of animal source food (>15% of dietary energy supply) increased from 33% to over 50%. While food supply has increased globally, food self-sufficiency (domestic production>2500 kcal/cap/d) has not changed remarkably. In the beginning of the study period insufficient domestic production meant insufficient food supply, but in recent years the deficit has been increasingly compensated by rising food imports. This highlights the growing importance of food trade, either for food supply in importing countries or as a source of income for exporters. Our results provide a basis for understanding past global food system dynamics which, in turn, can benefit research on future food security.

## Introduction

Feeding the world's population is a challenge that is likely to only deepen in the future. Global population is expected to reach 9 billion by 2050, adding two billion mouths to feed to the current population [1]. In many parts of the world, land and water resources needed for food production are already overexploited, questioning the sustainability of current use of natural resources in agriculture [2]–[5]. Furthermore, it is possible that changing climate will increase the scarcity of those critical resources [6], [7], as precipitation variability is projected to increase and droughts and floods are likely to become more frequent [8], [9]. Meanwhile, food consumption habits are changing: humans consume more calories than before, and diets consist increasingly of very resource intensive animal products [10]–[12]. It has been estimated that with current consumption, food production should roughly double by 2050 to meet the increasing demand [4], [13].

Substantial changes concerning global food supply have taken place in the past decades. During the past 50 years, the world's population has more than doubled [1] and at the same time food production practices have developed from traditional farming towards more intensive and industrialised production [14]. Increased globalisation has shaped the way and location of food production. Together with growing wealth [12], [15] and urbanisation [16] they have changed our consumption habits [17]. Thus, in a relatively short period of time, the way we feed the global population has changed tremendously.

Number of studies address the present situation and future projections of food availability [18]–[22]. In addition, the FAO (United Nations Food and Agricultural Organisation) has released a series of global food insecurity reports that date back to 1999 [23], but these, too, focus mainly on the present situation. Studies on the historical trends of food availability are mostly limited to various regional and case studies [24]–[26]. Thus far, the most comprehensive global assessment of past food availability is an FAO report by Alexandratos and Bruinsma [27], which analyses trends in food consumption and undernourishment during 1970–2006. Their study addresses global and regional trends, with a particular focus on developing countries, and thus lacks analyses at country level. Pradhan et al. [28] identified changes in dietary patterns in terms of food composition and energy content between 1961 and 2007. This study, however, focuses on greenhouse gas emissions embodied in different diets, ignoring further analysis of food availability.

To our best knowledge, more detailed global studies focusing on how food availability has developed during the past decades do not exist. Historical understanding of these past trends would, nevertheless, be vital for our efforts to tackle the food security challenges of the future. It is worth of addressing, however, that food security cannot be achieved by merely producing sufficient quantities of food. In addition to food availability, food security is generally considered to have three other dimensions: access (sufficient resources to obtain appropriate foods), utilisation (appropriate use based on knowledge of basic nutrition) and stability (of all these dimensions over time) [23]. In this paper we concentrate on food availability, while acknowledging the importance of the other three dimensions for a secure food system.

In this paper we thus aim to assess how global food availability has developed during the period 1965–2005. We quantify dietary energy supply and dietary energy production at national level for nine time steps during this period. Since growing share of agricultural products is not consumed at the place of production but traded elsewhere [29], we also investigate the trends in food self-sufficiency as well as the role of international food trade in improving food availability in the past. Producing the same dietary energy by animal source foods requires much more land and water resources than crop food production. To address the issue of efficiency of food supply, we look at whether diets have changed globally in this regard during the last decades.

## Materials

Our analyses of food availability, food self-sufficiency and food trade were based on the calculation of national level dietary energy supply (*DES*; measured in kcal/cap/d) and dietary energy production (*DEP*; kcal/cap/d). These were first calculated for individual food products in terms of mass (kg) and later converted into dietary energy (kcal). The calculations were performed for nine time steps during 1965–2005. In the following sub-sections we describe the data used in the analyses (see Table S3 in File S1 for a summary of data sources) and some basic operations performed on the extracted data, while the applied methods are described in the next section.

### Food supply quantity and food production quantity

Food supply quantities *FSQ* (kg), food exports *E* (kg), food imports *I* (kg) and changes in stocks *dS* (kg) were derived for 70 food crop and 24 animal source products from the FAO Commodity Balances database [30] (see details in Table S1 and related text in File S1). Data for 174 countries and the period 1965–2005 were extracted (see Table S2 and related text in File S1). To reduce inter-annual variation, we used five-year averages based on annual data for 1963–2007 (most recent available data at the time of the analysis), resulting in nine time steps (i.e. 1965, 1970, 1975, 2005).

Food production quantity FPQ (kg) of each product *i* was calculated for every country by using national data on food supply, exports, imports and stock variation (Equation 1). 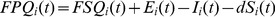
(1)


We decided not to use the production data provided by FAO in the same database directly, as those data also include crop production for other purposes than food supply for human consumption.

The above-described formula assumes that all exports, imports and goods adding to or derived from stocks could be used for human food supply. Therefore, any imports and stock withdrawals of human food products that are actually used for other purposes –mainly animal feed and further processing– decrease the FPQ. This illustrates the fact that when using food products for fodder or processing, calories readily available for human consumption are temporarily “lost” but later return to human use (although in lesser extent) as animal source foods or processed products. In a few cases a country's total food production (in terms of calories) was found to be negative, which normally indicates a very calorie-inefficient diet with a high consumption of animal products.

### Population data

National population data for each time step were derived from the FAOSTAT Population database [31], which is based on the 2010 Revision of the World Population Prospects from the UN Population Division [1].

### Filling of data gaps

Time series for 25 of the 174 countries were incomplete (see details in Table S2 and related text in File S1) due to historical changes in nations' existence. For example for the former Soviet Union nations, only the period after 1991 was available for individual countries. In these cases, product-specific food supply of the “mother country” on a given year was divided between the smaller, present-day nations in proportion to the smaller nation's share of the countries' combined supply in the earliest possible data year. For example, in the case of former Soviet Union countries, food supply of the Soviet Union in years 1965–1990 was divided between its post-Soviet states in proportion to each state's share of all the states' combined food supply in time step of 1995. In cases where this was not possible (combined supply of a certain product in 1995 was 0), supply in 1965–1990 was divided between post-Soviet states in proportion to their population in 1995.

The same procedure was followed with exports and imports. It should be noted that this method does not take into account any trade within the mother country. Thus, in the case of Russia, for example, export and import quantities in years 1965–1990 only represent trade flows from Russia to countries outside the Soviet Union or from non-Soviet countries to Russia. In some cases, this procedure may affect the FPQ calculations. However, sufficient data to estimate trade flows within the mother countries were not available.

### Dietary energy calculations

After compiling the database for FSQ and FPQ, these were converted into national level dietary energy supply *DES* (kcal/cap/d) and production *DEP* (kcal/cap/d) by using product and country specific conversion factors *c* (kcal/kg) and population data *P* (Equations 2 and 3). 
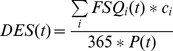
(2)

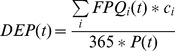
(3)


We calculated these conversion factors based on the FAO Food Balance Sheets [32], which provide country specific data for each analysed product both in kg and in kcal. We assumed that energetic values of food products do not change over time, and thus calculated the conversion factors using averaged data from the three latest data years (2005–2007). In cases where national conversion data were not available, we used product specific global averages. The converted values were then divided by the country's population on a given data year, in order to calculate the per capita dietary energy supply (DES, kcal/cap/d) and dietary energy production (DEP, kcal/cap/d) used in the analyses. National level DES and DEP for each time step can be found in Supporting Information (Data S1).

## Methods

### Food availability

**Figure 1:**
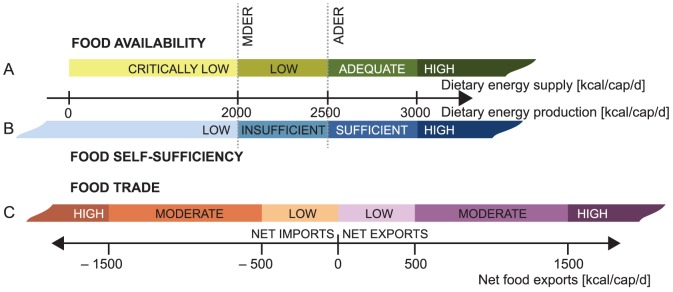
Classification of food availability (A), food self-sufficiency (B) and food trade (C). MDER and ADER refer to minimum dietary energy requirement and average dietary energy requirement, respectively (see Methods section and the FAO [33]).

In this study, we measured the level of food availability by looking at the national average DES (i.e. dietary energy supply, see Data S1 for national level data) relative to dietary energy requirements. We based our classification on global averages of Minimum Dietary Energy Requirement (MDER) and Average Dietary Energy Requirement (ADER), defined by FAO for years 1990–2005 [33]. Global averages over 1990–2005 of MDER (1820 kcal/cap/d) and ADER (2200 kcal/cap/d) were considered as the thresholds for critically low food supply and low food supply respectively. However, the used FAO food supply data [30] do not refer to the actual consumption of food but include also the wastage of food during distribution and consumption phases of food supply chain (the other losses are taken into account in the database). Based on Gustavsson [34] and Kummu et al [35], we estimated this food waste to be approximately 12% of food supply (in terms of kcal) globally. This percentage currently varies across countries between 5–21%. However, the fraction of waste in different countries has also varied a lot historically, and as reliable historical data are not available on a country scale, we decided to use the current global average value of 12% to adjust the MDER and ADER thresholds. Basically, this means that the thresholds used in the analysis may be slightly too high for countries with low waste percentage, and on the other hand, too low for countries with high waste percentages. The adjustment of MDER and ADER resulted in the following classification of food availability (see also Figure 1A):

- Critically low supply: DES of less than 2000 kcal/cap/d
- Low supply: DES of 2000–2500 kcal/cap/d
- Adequate supply: DES of 2500–3000 kcal/cap/d
- High supply: DES of over 3000 kcal/cap/d

Threshold value for high food supply corresponds to a food consumption level that is higher than any national ADER during the reference period [33].

There are two basic strategies to maintain a sufficient level of food supply: a) to produce food domestically with own resources, i.e. food self-sufficiency, or b) to benefit from food trade (exporters by creating income from food exports and importers by relying on external resources for food production). Thus, in addition to analysing food availability, we looked at the countries' level of food self-sufficiency and food trade. In the following sub-sections we describe the methods used in these analyses.

### Food self-sufficiency

Food self-sufficiency was defined here as country's domestic food production in relation to the statistical food supply requirements of its population. It was measured by comparing the country's DEP (i.e. dietary energy production, see Data S1 for national level data) with global ADER (i.e. average dietary energy requirement). Food self-sufficiency was analysed using the same thresholds that were defined for analysing food availability level (see also Figure 1B):

- Low production: DEP of less than 2000 kcal/cap/d
- Insufficient production: DEP of 2000–2500 kcal/cap/d
- Sufficient production: DEP of 2500–3000 kcal/cap/d
- High production: DEP of over 3000 kcal/cap/d

### Food trade

Our food self-sufficiency indicator illustrates the share of domestic food production of a country's statistical food supply requirements. However, it does not consider its capacity to produce its reported DES [30], [32] domestically. Therefore, we also looked at the national food supply-production balance by calculating the difference between DES and DEP (see Data S1 for trade data). It should be noted that negative and positive supply-production balances are not synonymous terms for net exports and net imports, as they also include the variation in stocks. In most cases, however, the role of stock variation is insignificant compared to imports and exports, thus this balance mainly illustrates the importance of food trade for sustaining a specific DES. The following classification was used to analyse food trade (see also Figure 1C):

- High net imports: DES – DEP of over 1500 kcal/cap/d
- Moderate net imports: DES – DEP of 500–1500 kcal/cap/d
- Low net imports: DES – DEP of 0–500 kcal/cap/d
- Low net exports: DEP – DES of 0–500 kcal/cap/d
- Moderate net exports: DEP – DES of 500–1500 kcal/cap/d
- High net exports: DEP – DES of over 1500 kcal/cap/d

Thresholds of 500 and 1500 kcal/cap/d correspond to 20 and 60% of global ADER respectively. This means that a country with high net exports (over 1500 kcal/cap/d) could, in theory, provide for over 60% of the adequate nutrition of a nation of equal population.

### Composition of diets

Apart from the sheer energy content of food, we also analysed the composition of diets during the study period, specifically the share of animal based calories of total DES (see Data S1 for national level data). Although animal products are not necessarily needed to maintain a healthy life, they are often seen as the easiest way to ensure right protein intake, as they include adequate amounts of all the amino acids essential for humans [36], [37]. Thus, to ensure sufficient intake of all these amino acids, we considered here that some amount of animal products would be needed for a balanced diet. However, in many parts of the world the consumption of animal products is multifold compared to these requirements [10], [38]. While higher consumption of animal products may not be a health risk, animal source food production uses land and water resources less efficiently than crop food production for direct human consumption [39], [40]. The thresholds we used for different levels of supply of animal products follow the thresholds suggested by Smil [10] and Falkenmark and Lannerstad [41]:

- Inadequate: 0–5% of total DES
- Adequate: 5–15% of total DES
- High: 15–25% of total DES
- Very high: >25% of total DES

## Results

### Food availability

**Figure 2:**
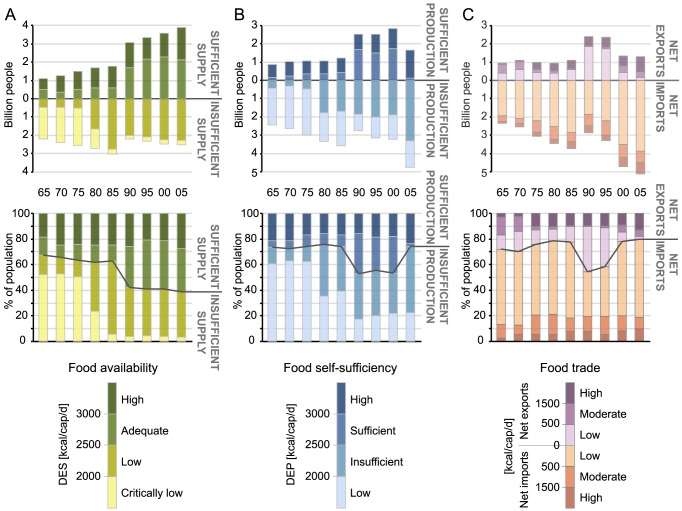
Global population in different food availability (A), food self-sufficiency (B) and food trade (C) categories. Upper figures express the population in absolute numbers and lower figures in relative numbers. DES and DEP refer to dietary energy supply and dietary energy production.

Our analysis indicates that global food availability has improved substantially both in absolute and relative terms during the study period (1965–2005). The percentage of population living in countries with sufficient food supply (>2500 kcal/cap/d) has almost doubled from the 33% in 1965 to 61% in 2005 (Figure 2A). In absolute terms the increase is even more considerable; from 1.1 billion people in 1965 to 3.9 billion in 2005. While the share of population with insufficient food supply (<2500 kcal/cap/d) has nearly halved, the share of the most vulnerable group, those living with less than 2000 kcal/d, has dropped from 52% to 3%. In absolute terms, however, population with insufficient food supply was even slightly bigger in 2005 than it was in the beginning of the reference period (Figure 2A). Global per capita dietary energy supply has increased by 20% since 1965, which means that in absolute terms it has more than doubled.

**Figure 3:**
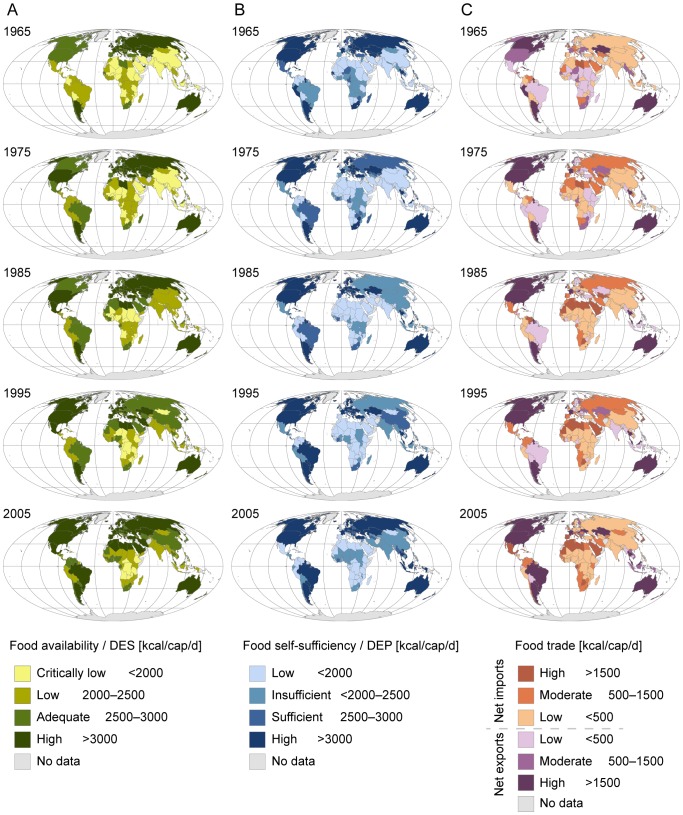
Food availability (A), food self-sufficiency (B) and food trade (C) mapped for five time steps. DES and DEP refer to dietary energy supply and dietary energy production.

Largest improvements in food availability can be seen in the MENA region, Latin America and China (Figure 3A). While China has risen from critically low food supply to adequate supply, in some Latin American countries, particularly Brazil and Mexico, dietary energy supply has increased from insufficient levels to over 3000 kcal/cap/d. The same kind of development can be seen in the MENA region, where by 2005 almost all countries had a dietary energy supply of over 3000 kcal/cap/d. There has been some improvement in Sub-Saharan Africa, particularly in Western Africa, but most countries are still below the threshold of sufficient food supply. In North America, Russia and most European countries, food availability has stayed in sufficient levels during the whole study period.

### Food self-sufficiency

The percentage of global population living in food self-sufficient countries has remained relatively stable during the study period. Apart from 1990–2000 when the figure rose to about 45%, these countries have accounted for about 25% of the global population (Figure 2B). While food self-sufficiency has not increased considerably, the share of population in the lowest production class has decreased notably since 1965. Meanwhile, the percentage of population producing 2000–2500 kcal/cap/d has increased, suggesting that many countries have risen from the low production category to the next one (i.e. insufficient production one). The percentage of population in the high production class has not changed considerably. The self-sufficient class peaked in 1990 before declining to very low numbers by 2005.

There are a number of countries, e.g. Brazil and Southeast Asian countries like Indonesia, Malaysia and Vietnam, that have moved from lower DEP to very high DEP (Figure 3B). However, there is also a tendency of a dramatic decline in food self-sufficiency in many countries, such as South Africa and Zimbabwe in Africa, Mexico and Peru in Latin America and Mediterranean countries like Italy and Spain.

**Figure 4:**
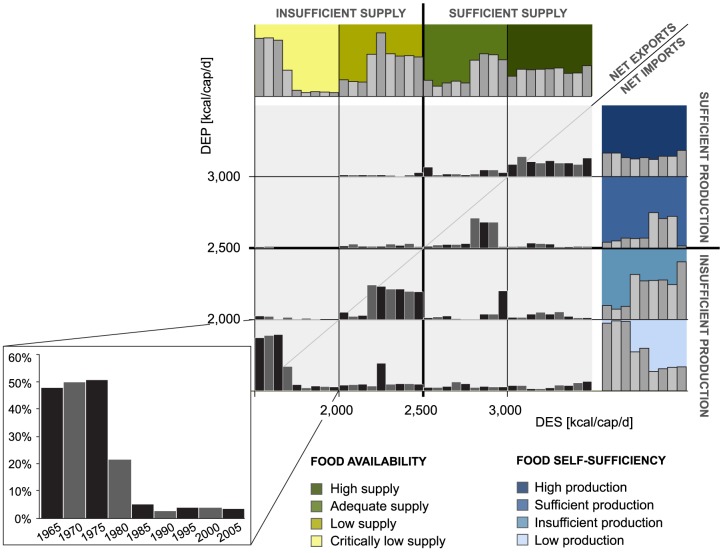
Share of global population living in different food availability (x-axis; measured with DES – dietary energy supply) and food self-sufficiency (y-axis; measured with DEP – dietary energy production) categories during the study period. Bar charts in the end of rows and columns illustrate the sum of each row/column.

When DEP was compared with DES (Figure 4), we found that throughout the reference period, the majority of world's population has been living in countries where DES and DEP are quite well in balance. As expected, most countries with sufficient dietary energy production also have a sufficient food supply (upper right categories of Figure 4), suggesting that food is produced primarily to secure domestic food supply and only secondarily for export. In the beginning of the study period, a vast majority of the population in countries with insufficient DEP also had an insufficient food supply (lower left categories of Figure 4), indicating that these countries were not able to import food to secure domestic supply. The incremental increase of population in the lower right categories of Figure 4 (DES>2500 kcal/cap/d and DEP<2500 kcal/cap/d) suggests that during the study period the dependence on food imports has increased somewhat.

### Food trade

The majority of global population has been living in net food import countries throughout the study period. In 1965, these countries accounted for 72% of the population (Figure 2C). The figure fell to under 60% for the period between 1990 and 1995, but otherwise has been rising slowly, reaching 80% in 2005. In absolute terms this means that the population living in net importing countries has more than doubled from the 2.4 billion in 1965 to 5.1 billion in 2005. However, a large majority of this population lives in countries where net imports are low (less than 500 kcal/cap/d).

Although the population importing over 500 kcal/cap/d has not seen a very large relative increase (from 13% in 1965 to 19% in 2005), there has been a substantial increase in the highest import category. The population of countries that are importing over 1500 kcal/cap/d has more than tripled from 3% (less than 0.1 billion) to 10% (over 0.6 billion), which in absolute terms equals a 7-fold increase. Similar trend has been even stronger in the net exporter side. While the percentage of population in net exporting countries has declined during the reference period, the percentage of highest exporters (over 1500 kcal/cap/d) has increased over fourfold from 3% in 1965 to 13% in 2005. In absolute terms this equals a 9-fold increase in population from less than 0.1 billion to over 0.8 billion.

The importance of imports has notably increased in the MENA region, Southern Africa, Central America and Southern Europe (Figure 3C). In the case of Southern European countries, this is partly due to their specialisation in the production and export of fruits and vegetables, while cereals, which account for a much larger share of total dietary energy, flow in the opposite direction. In Sub-Saharan Africa, a remarkable change can be seen in most countries, moving from low/moderate exporters to low/moderate importers. A few countries, namely Australia, Argentina, Canada and USA have dominated global food exports throughout the study period, but in recent years Brazil and many Southeast Asian countries have also increased their food production for export.

### Composition of diets

**Figure 5:**
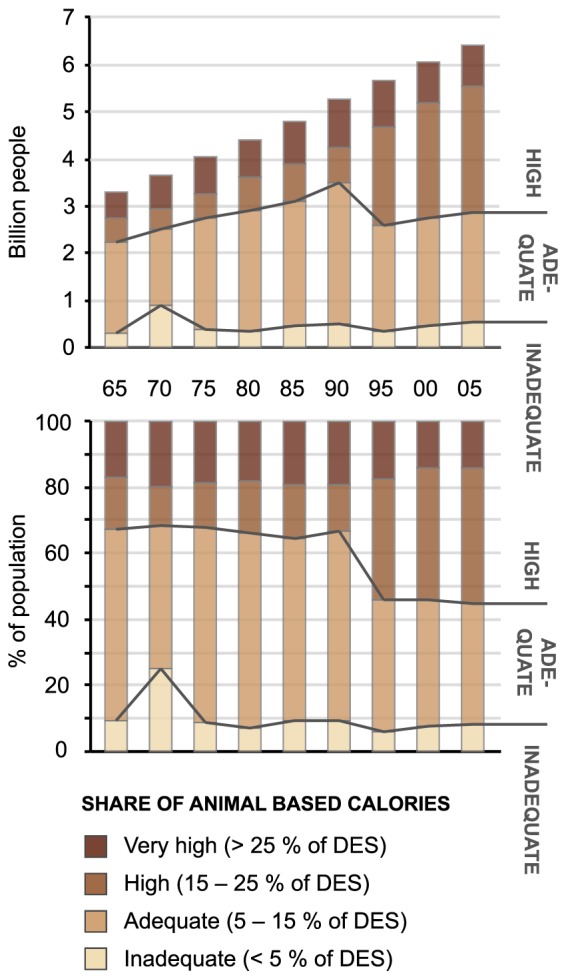
Global population living in different categories based on supply of animal based calories. Upper figure expresses the population in absolute numbers and lower figure in relative numbers.

As global food availability has improved in terms of dietary energy supply, the composition of diets has also changed considerably. In the beginning of the reference period, majority of the world's population (58%) got 5–15% of their energy supply from animal source foods, i.e. had adequate share of animal products (Figure 5). However, almost all of this population was living in countries with insufficient food supply (DES of less than 2500 kcal/cap/d, see Figure S1 in File S1). By 2005 over a half of the world's population lived on a very resource intensive animal source food based diet (>15% of dietary energy intake from animal source foods), while in 1965 the percentage was 33%. Taking into account the overall increase in food supply, this means that in absolute terms, the supply of animal based calories has increased 2.6 fold.

## Discussion

Our results indicate that while food availability improved globally during the reference period (1965–2005), food self-sufficiency (dietary energy production of >2500 kcal/cap/d) did not change a lot (see Figure 2). In the beginning of the study period insufficient domestic production meant insufficient food supplies. In recent years the production deficit has been compensated more and more by increased food imports. Indeed, the trends we observed indicate that food trade has become more important during the study period. An increasingly large population is dependent on food trade, either as food supply in importing countries or as a source of income in exporting countries. Similar trends have been found in previous studies. Alexandratos and Bruinsma [27], for example, report a substantial increase in per capita food consumption (in terms of dietary energy) in all regions of the world during 1970–2006. International trade statistics by WTO [42], on the other hand, show an almost twofold increase in global per capita exports of agricultural products (in terms of weight) during 1965–2005. However, increased food trade does not improve food availability for all. When comparing the per cap GDP (derived from UN [43]) in different food availability and food production classes (see Figure 4), we found that average per cap GDP in countries that achieve sufficient food supply by imports was approximately tenfold compared to countries with insufficient food supply and production. Thus, although rising food imports seem to have improved food availability globally, securing food supply by imports appears to require a strong enough economy.

Imports of food are not merely a way to secure food supply but increasingly also a means to maintain a certain lifestyle and diet. In recent years many large importers live on a very high calorie diet (>3000 kcal/cap/d) with over 15%, or in some countries even over 25%, of calorie intake from animal source foods. The results of higher dietary energy intake can be seen as an increased problem of obesity and overweight all over the world [44]. The increasing trend in animal source food supply has previously been observed by Kastner et al. [45] and Pradhan et al. [28], and is particularly worrying for the (future) use of natural resources, as animal source food production requires intensive use of e.g. land and water resources [41].

### Drivers behind the findings

In order to identify possible drivers for the trends presented above, we conducted a correlation analysis between DES (i.e. dietary energy supply), DEP (i.e. dietary energy production), food trade, animal source food supply, one development variable (per cap GDP – gross domestic product) and two variables related to resource availability (freshwater availability per capita and arable land per capita). Time series for these variables were derived from various UN databases [43], [46], [47]. Correlations were calculated country-wise over the study period (9 time steps). In the case of GDP, data were available from the year 1970 onwards, thus these analyses were conducted over 8 time steps. Due to poor data availability, countries with incomplete time series had to be excluded from the analyses, resulting in 145 countries for GDP, 135 countries for water availability and 146 countries for land availability analyses.

**Figure 6:**
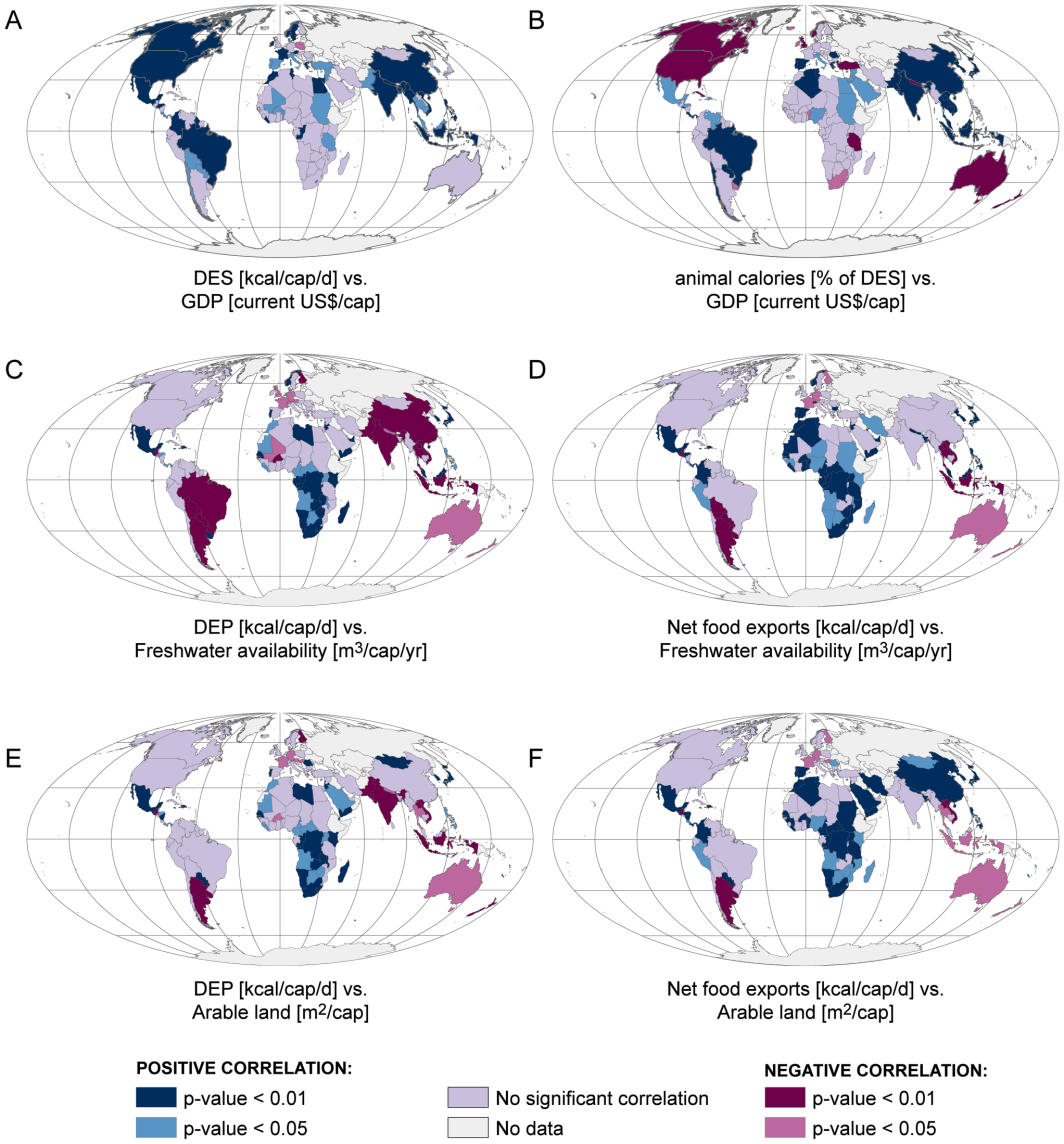
Results of correlation analyses mapped. A: GDP (gross domestic product) and DES (dietary energy supply); B: GDP and supply of animal based calories; C: freshwater availability and DEP (dietary energy production); D: freshwater availability and net food exports; E: arable land and DEP and F: arable land and net food exports. See Table 1 for tabulated population results.

We found that DES tends to increase when income rises (Figure 6A, Table 1). Countries where GDP and DES had a significant positive correlation (*p*<0.05) represented 72% of the world's population in 2005. For 61% of the population, including e.g. China, India and United States, correlations were even highly significant (*p*<0.01). Significant positive correlations (*p*<0.05) between GDP and the supply of animal based calories were found in countries that in 2005 accounted for 64% of the world's population (Figure 6B, Table 1). For a vast majority of these countries (representing 58% of total global population) correlations were highly significant (*p*<0.01). These countries include many newly industrialised countries, such as Brazil, China and India. Interestingly, in some countries, particularly highly industrialised countries like Australia, UK and US, we found significant negative correlations between GDP and the supply of animal based calories (Figure 6B). This suggests that in some countries with high GDP, the share of animal based calories actually decreases when income rises.

**Table 1:** Results of correlation analysis.

	Significance level	DES vs. GDP	Animal based calories vs. GDP	DEP vs. freshwater availability	Net food exports vs. freshwater availability	DEP vs. arable land	Net food exports vs. arable land
Positive correlation	*p*<0.01	61% (3994)	56% (3632)	9% (580)	17% (1133)	9% (607)	39% (2549)
	0.01≤*p*≤0.05	10% (681)	8% (518)	4% (236)	6% (408)	8% (550)	5% (312)
Negative correlation	*p*<0.01	0% (0)	9% (565)	51% (3321)	7% (486)	26% (1679)	2% (144)
	0.01≤*p*≤0.05	1% (38)	1% (66)	4% (285)	3% (173)	4% (293)	7% (477)
No significant correlation	*p*>0.05	19% (1268)	18% (1202)	24% (1579)	58% (3801)	44% (2873)	39% (2520)
No data		8% (497)	8% (497)	7% (478)	7% (478)	7% (477)	7% (477)

^6^) are in parentheses. DES stands for dietary energy supply (kcal/cap/d), GDP for gross domestic product (current US$/cap) and DEP for dietary energy production (kcal/cap/d). Units of freshwater availability, net food exports and arable land are m^3^/cap/yr, kcal/cap/d and m^2^/cap, respectively. Share of global population in 2005 in countries with significant positive and negative correlations. Absolute population numbers (x10

Although correlations between resource availability and food production and trade were discovered, they seem to be specific to certain regions. We found significant positive correlations between resource availability and both DEP and net food exports in many parts of Africa and Central America (Figures 6C–6F). Significant positive correlations (*p*<0.05) between net food exports and arable land were the most widespread of these, including also China and other Asian countries and accounting for 44% of the world's population (Table 1). As most of these countries are net importers (Figure 3C), the positive correlations suggest that in these areas food production might be limited by resource constraints and thus compensated with food imports. However, it is also possible that domestic food production has simply not kept up with the increasing supply demands from growing population and changing diets. In Mexico, for example, per capita freshwater and land resources are still quite abundant despite the rapid population growth. Yet, in recent years it has become a high net importer of food (see Figure 3C), with over 1800 kcal/cap/d imported in 2005. In a number of countries (e.g. Argentina, Australia and India) we also found significant negative correlations between resource availability and DEP and/or net food exports (Figures 6C–6F). For DEP and land/water availability, significant negative correlations (*p*<0.05) were found in countries that represent 30% and 55% of global population, respectively (Table 1). This indicates that in these countries, agricultural production and trade patterns are driven by other factors than availability or water and land resources.

Thus, although the relationship between food availability and economic development seems fairly straightforward, the dynamics behind food availability, food production and trade are still not very well understood, as suggested by many. For example, in their global analysis Kumar and Singh [48] found no clear relationship between the relative availability of water resources and imports of food. In some cases scarcity of resources can even be intensified by the production of agricultural products for export, as was found by Porkka et al. [49] in the case of the Aral Sea region. On the other hand, Fader et al. [50] found recently that a number of countries rely on food imports due to land and water constraints on domestic food production, and that this number is likely to increase in the future. However, they also note that in many countries high imports are not due to resource constraints but have other, e.g. economic, drivers.

### From food availability to food security

As mentioned in Introduction, food security cannot be accurately analysed merely based on the sheer quantity of food. Other dimensions of food security include access to food, utilisation of food and stability of food secure conditions over time [23]. While the latter two dimensions are difficult to acknowledge in quantitative analyses with current data, access to food has been incorporated in the FAO food security studies in the past [51]. Recently, the FAO released a new set of food security indicators for the years 1991–2011 [33]. We used these data, specifically the prevalence of food inadequacy indicator, to analyse the differences in food security within countries. This indicator is a less conservative measure of the occurrence of food insecurity than the previous prevalence of undernourishment, which is the traditional FAO estimator for chronic hunger.

**Figure 7:**
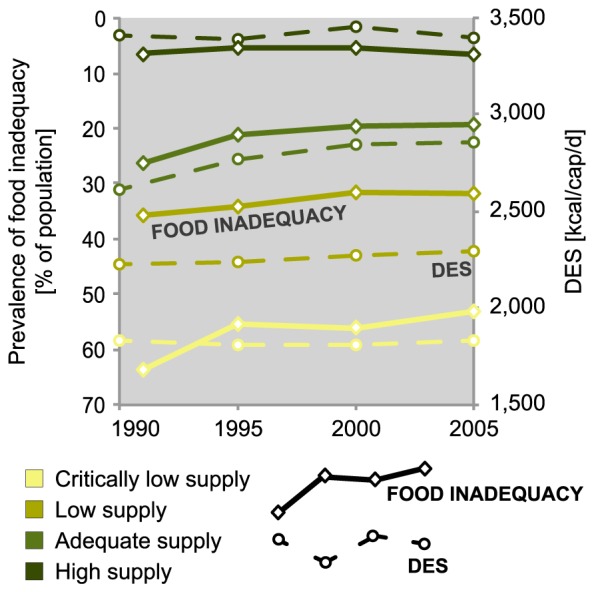
Trends in dietary energy supply (DES) and prevalence of food inadequacy [33] in different food security classes during 1990–2005. As food inadequacy data for 1990 were not available, data for 1991 were used here instead. Note: The FAO does not report prevalence of food inadequacy numbers that are under 5%, thus the actual numbers in the high supply category are likely to be even lower.

To analyse the prevalence of food inadequacy in different food availability classes, we calculated population-weighted averages of DES (i.e. dietary energy supply) and prevalence of food inadequacy in each class for years 1990 (due to lack of earlier data, 1991 for food inadequacy), 1995, 2000 and 2005. As expected, the prevalence of food inadequacy tends to be lower in countries with high average DES and higher in countries with low average DES (Figure 7). This tendency can also be seen when looking at trends during 1990–2005. In countries with adequate and low food supply, average DES increased during the 15-year period, while prevalence of food inadequacy decreased. In countries with high food supply, both average DES and prevalence of food inadequacy remained relatively stable. However, in countries with critically low food supply the prevalence of food inadequacy decreased from 64% to 53%, while average DES did not change substantially. Although smaller prevalence of food inadequacy can generally be considered positive, this finding suggests that in these countries, inequality regarding the distribution of food supply increased during the reference period. In 2005, for example, almost a half of the population in countries with critically low food supply actually received adequate energy supply, which indicates that those that did not were likely to be severely undernourished. On the other hand, in countries with a very high average DES (over 3000 kcal/cap/d), the average prevalence of food inadequacy stayed well below 10%, suggesting that the national DES was distributed quite equally.

### Limitations and way forward

Our food availability analysis focuses on the supply of dietary energy, thus ignoring the shares of different elements of food supply (carbohydrates, proteins, fats, vitamins etc.) that are required for a healthy and balanced diet. Differentiating between all these elements would have added another level of complexity to the assessment. Instead, we chose to include a simple analysis of diet composition by looking at the share of animal based calories of total dietary energy supply. Although this analysis does not attempt to give a detailed picture of diet composition, it manages to capture two important aspects of animal source foods: Firstly, as animal source foods are the simplest way for humans to ensure the intake of all the essential amino acids, we considered that some level of animal products is needed for a balanced diet. This is taken into account when defining the thresholds for different classes of animal source food supply (see the Methods section). Secondly, production of animal source foods is drastically different from that of food crops in terms of efficiency (of e.g. the use of energy and land and water resources, see FAO [40] and González [52]). Thus, our food composition analysis gives insight also on the environmental consequences of different diets. However, it should be noted that not all agricultural land used for animal source food production (e.g. much of pasture land) could be converted into cropland. Thus, in those cases reducing animal source food production might not increase the efficiency of dietary energy production. Furthermore, we did not differentiate between aquatic and terrestrial animal source foods whose production is fundamentally different with regard to resource use. Thus, any further analysis of the environmental aspects of different diets should take these differences into account.

As discussed in the previous sub-section, the dynamics behind the trends we observed are still not well understood. Although economic development and land and water resource constraints seem to explain some of the trends, they clearly do not reveal the whole picture. One major factor contributing to the developments we observed is urbanisation. Although globally a considerable proportion of urban dwellers are undernourished, urban diets as a whole tend to change towards higher consumption of animal products, "luxury" foods and overall calories [16], [53]. Furthermore, growing urban areas and populations effectively mean less local production [54]. These two factors combined contribute to food trade dependency in a very obvious way. In addition to urbanisation, free markets promote the importance of food trade by allowing and making it profitable for countries to specialise in the production and export of certain food products while meeting the demand of others by imports (as was discussed in Results section regarding Southern Europe). With regard to resources contributing to food production, limited land and water resources are not the only possible production constraints. For example energy, fertilizers and labour are all obvious inputs of agricultural production that we did not include in this study. In addition to resources, climatic factors, such as growing season length, affect countries' food production capacity. Thus, in order to fully understand the dynamics behind global food system, we suggest that future research should address the possible drivers more thoroughly.

Because of data limitations, the calculation of population (or shares of global population) in different categories of food availability, food self-sufficiency, food trade or composition of diets is based on a categorization at country level and neglects therefore the distribution and variability within countries. We are aware of the large differences in food availability depending on per capita income, availability of imported food in urban or rural areas and composition of diets, which can vary a lot even between members of the same household. The main objective of this study is to identify long-term trends at global scale. Findings for specific countries are limited to country averages and interpretation of the results at country level would benefit from further disaggregation by regional case studies.

## Conclusions

In this article, we investigated past global trends in food availability by quantifying country-level dietary energy supply and production during 1965–2005. Further, we examined how food self-sufficiency has developed globally, and analysed the role of food trade in improving food security. We found that food availability has improved considerably while food self-sufficiency has remained relatively low during the entire study period. Trade of food products has, thus, soared in importance in securing an adequate food supply. In many parts of the world, diets are increasingly abundant in calories and animal source foods.

Within the past 50 years, the world has thus moved from food insufficiency towards an increasing dependency on food trade. This has improved food availability, but mainly in regions with a sufficiently strong economy to be a notable player in the trade markets. While a secure food supply has been intentionally outsourced in various parts of the globe, a large share of global population is still living with insufficient food supply. Food security is not merely a question of food availability but increasingly also a question of access to food. At global scale food supply would be sufficient to feed the entire population but its uneven distribution leaves a notable proportion of population food insecure while others live in abundance of food. Thus, while global food supply could be increased by e.g. novel technological solutions, reform of current agricultural practices and reduction of food waste [4], [55], any substantial improvement in food security will require real efforts for a more equal distribution of global food supply.

## Supporting Information

File S1 **Supporting information on materials used and results obtained, containing Tables S1, S2 and S3, and Figure S1.** Table S1, Food products included in this study. Table S2, Data availability of countries included in the analysis. Table S3, Sources and description of materials used in the analysis. Figure S1, Share of global population living in different food availability (x-axis; measured with DES - dietary energy supply) and diet composition (y-axis; measured with the share of animal based calories of total DES) categories during the study period.
(TIF)

Data S1 **National level data on dietary energy supply (DES), dietary energy production (DEP), net food exports and animal based calorie supply.**
(XLSX)
